# Homocysteine Restricts Copper Availability Leading to Suppression of Cytochrome C Oxidase Activity in Phenylephrine-Treated Cardiomyocytes

**DOI:** 10.1371/journal.pone.0067549

**Published:** 2013-06-20

**Authors:** Xiao Zuo, Daoyin Dong, Miao Sun, Huiqi Xie, Y. James Kang

**Affiliations:** 1 Regenerative Medicine Research Center, West China Hospital, Sichuan University, Chengdu, Sichuan, China; 2 Department of Pharmacology and Toxicology, University of Louisville School of Medicine, Louisville, Kentucky, United States of America; Auburn University, United States of America

## Abstract

Cardiomyocyte hypertrophy induced by phenylephrine (PE) is accompanied by suppression of cytochrome c oxidase (CCO) activity, and copper (Cu) supplementation restores CCO activity and reverses the hypertrophy. The present study was aimed to understand the mechanism of PE-induced decrease in CCO activity. Primary cultures of neonatal rat cardiomyocytes were treated with PE at a final concentration of l00 µM in cultures for 72 h to induce cell hypertrophy. The CCO activity was determined by enzymatic assay and changes in CCO subunit COX-IV as well as copper chaperones for CCO (COX17, SCO2, and COX11) were determined by Western blotting. PE treatment increased both intracellular and extracellular homocysteine concentrations and decreased intracellular Cu concentrations. Studies *in vitro* found that homocysteine and Cu form complexes. Inhibition of the intracellular homocysteine synthesis in the PE-treated cardiomyocytes prevented the increase in the extracellular homocysteine concentration, retained the intracellular Cu concentration, and preserved the CCO activity. PE treatment decreased protein concentrations of the COX-IV, and the Cu chaperones COX17, COX11, and SCO2. These PE effects were prevented by either inhibition of the intracellular homocysteine synthesis or Cu supplementation. Therefore, PE-induced elevation of homocysteine restricts Cu availability through its interaction with Cu and suppression of Cu chaperones, leading to the decrease in CCO enzyme activity.

## Introduction

Disturbance in the mitochondrial structure and metabolism makes a critical contribution to the pathogenesis of hypertrophic cardiomyopathy [1]–[3]. Among the essential components involved in the mitochondrial integrity and function, cytochrome c oxidase (CCO) is the last of the three proton-pumping assembles of the mitochondrial respiratory chain, catalyzing the transfer of electron from reduced cytochrome c to molecular oxygen, the final electron acceptor. Both experimental and clinical studies demonstrated the determinant role of CCO depression in the initiation and progression of cardiac hypertrophy and dysfunction [4]–[6].

CCO is composed of 13 subunits, three of which (COX-I, -II, and -III) are encoded by the mitochondrial DNA and the rest are encoded by the nuclear DNA [7]–[9]. In these subunits, COX-I and COX-II contain copper (Cu) active sites and constitute the catalytic core of CCO [10]. In rodent models, Cu deficiency decreases the level of CCO subunit proteins and inhibits the CCO activity [11]–[13]. Cu chaperones for CCO, including cytochrome c oxidase assembly homolog 17 (COX17) and 11 (COX11), cytochrome oxidase-deficient homolog 1 (SCO1) and 2 (SCO2), are responsible for delivering Cu to the Cu active sites Cu_A_ on COX-II and Cu_B_ on COX-I in CCO [14]–[17]. Mutations in either SCO1 or SCO2 cause severe CCO assembly impairment and thus decrease the CCO activity [18], [19]. Loss of function of COX17 attenuates the delivery of Cu to SCO1 and COX11, and inhibits the CCO activity [20], [21]. Mutations in COX11 also suppress the CCO activity [22].

Cu concentrations decrease in pressure overload-induced hypertrophic hearts [23]. Dietary Cu supplementation restores Cu levels in the heart, recovers the CCO activity, and reverses hypertrophic cardiomyopathy in mouse model subjected to pressure overload [23]. In primary cultures of neonatal rat cardiomyocytes, exposure to phenylephrine (PE) causes depression in the CCO activity and cell hypertrophy. Addition of Cu to the culture recovers the CCO activity and reverses the hypertrophy [24]. It is thus concluded that the limitation of Cu availability to the CCO under the condition of either pressure overload [23] or PE treatment [24] is responsible for the depressed CCO activity. However, the mechanism by which Cu restriction takes place in the hypertrophic cardiomyocytes remains elusive.

Pressure overload increases cardiac homocysteine production [25]. Experimental and clinical studies produced supporting evidence that elevated blood levels of homocysteine are linked to increased risk of cardiovascular diseases [26]–[28]. The increase in the blood concentrations of homocysteine is associated with an increase in blood Cu concentrations [27]–[29]. Studies *in vitro* found that Cu and homocysteine form complexes, which elicit remarkable changes in the atherogenic activity in the cultured endothelial cells [30]–[32].

The present study was undertaken to specifically address the link between homocysteine and the restriction of Cu availability to CCO in PE-induced hypertrophic cardiomyocytes. To provide a comprehensive understanding, the effect of Cu restriction on Cu chaperones for CCO and on a critical subunit of CCO assembly, COX-IV, was determined. The results demonstrated that the interaction between Cu and homocysteine and the depression of Cu chaperones for CCO restrict the availability of Cu to the CCO, leading to the suppression of its enzymatic activity.

## Methods

### Cell Culture

Primary cultures of neonatal rat cardiomyocytes were established according to a procedure published previously [33]. Briefly, the hearts exercised from 1–3 day-old Sprague–Dawley rats were cut into 1.0 mm and digested with collagenase II (GIBCO, USA) and Trypsin (TN, GIBCO, USA) for 5 min, repeating 6 times. The animal procedure was approved by the Institutional Animal Care and Use Committee of Sichuan University. The heart tissue digests were suspended in H-DMEM (GIBCO, USA) supplemented with antibiotics (GIBCO, USA) and 10% fetal bovine serum (FBS, Hyclone). Fibroblasts were removed by pre-incubation for 1 h at 37°C. The cardiomyocytes in suspension were collected and seeded in 6-well-plate containing H-DMEM supplemented with 10% FBS, BrdU (Roche, USA) and antibiotics for 24 h before subsequent experiments. The purity of cardiomyocytes in cultures was determined by flow cytometry quantitative analysis of the cells labeled with fluorescent antibody for α-sarcomeric actin (Santa Cruz, USA), which was more than 90%.

### Experimental procedure

Cardiomyocyte hypertrophy was induced according to a previous publication [34], with some modifications. Briefly, primary cultures of neonatal rat cardiomyocytes were treated with phenylephrine (PE, Sigma-Aldrich) at a final concentration of 100 µM in cultures for 48 h in serum-free media, and then CuSO4 was added directly into the antibiotic-free cultures at a final concentration of 5 µM element in cultures for 24 h. At the end of the treatment, the cells were collected by trysinization, suspended in PBS buffer, and counted using a hemocytometer prior to subsequent analyses.

### Determination of Cu concentrations

Intracellular Cu concentrations were determined with graphite furnace atomic absorption spectrophotometer (AAS). Briefly, cells were collected by cell scraper, washed tree times with phosphate buffered solution and centrifuged at 500× g for 15 min. The precipitation was freeze-dried and dissolved in 50 µl concentrated HNO_3_ for 3 days. Finally, the prepared sample was dissolved in 0.5 ml de-ionized water, and then subjected to AAS analysis.

### Measurement of homocysteine concentrations

Homocysteine was measured by using HPLC as described earlier [35]. HPLC analyses were performed using Waters Millenium system (Waters 600) with a Waters 474 Fluorescent Detector and a Supelcosil LC 18 DB analytical column (250 mm×4.6 mm, 3 µM particle size). The temperature inside the column was maintained at 25°C during elution. The sample preparation was as follows: an aliquant of 90 µl cell lysate and 10 µl reducing agent (10% tris (2-carboxyethyl) phosphine hydrochloride, TCEP, Sigma) were mixed and incubated for 30 min at 4°C. The precipitation of proteins was achieved by addition of 100 µl methanol and subsequent centrifugation for 10 min at 2000× g. To 100 µl of supernatant, the following were added: 250 µl of borate buffer (0.125 M) containing EDTA (4 mM) adjusted to pH 9.5 and 10 µl of NaOH (1.55 M), and 10 µl of 7-fuorobenzo-2-oxa-1,3-diazole-4-sulphonic acid (SBD-F, 10 mg/ml solution in 0.125 M borate buffer, pH 9.5). The mixture was incubated for 60 min at 60°C and subjected to HPLC separation and analysis. The HPLC was performed as follows: mobile phase was 0.05 M KH_2_PO_4_, adjusted pH to 2.1 with ortho-phosphoric acid and 7% acetonitrile; flow-rate was 1.0 ml/min; and total elution time was 20 min. Fluorescent was monitored at 515 nm and excitation wavelength of 385 nm. Calibration was based on external standard using homocysteine diluted in distilled water and peak area was used for calculation of concentrations.

### The CCO activity

Mitochondria were isolated from collected cells using a mitochondrial isolation kit (Pierce, #89801) following the instruction provided. Bio-Rad protein assay was used to measure mitochondrial protein concentrations. An aliquot of 0.25 mg mitochondrial protein was prepared in 0.2 ml of isosmotic medium containing 10 mM KH_2_PO_4_, pH 6.5, 50 mM KCl, 0.25 M sucrose, 1 mg/ml BSA, and 2.5 mM n-dodecylmaltoside. The CCO activity was determined by adding 0.2 mM ferrocytochrome c. The enzyme activity was calculated from the rate of decrease in absorbance of reduced cytochrome c at 550 mM (ε  =  19.1 mmol-1cm-1). Because accurate estimation of the CCO activity could be compromised by variations in mitochondrial yield and integrity, the CCO activity in the present study was normalized using an invariant marker of mitochondrial enzyme activity, citrate synthase. Citrate synthase was assayed by following the reduction of 0.1 mM 5,5′- dithio-bis (2-nitrobenzoic acid) in the presence of 0.25 mg mitochondrial protein, 0.25 mM acetyl-CoA, and 0.5 mM oxalacetid acid in a medium with 40 mM PB, pH 8.0, 2 mM EDTA, 1 mg/ml BSA, and 0.1% Triton X-10. The change in the absorbance at 412 nm (ε = 13.6 mmol-1cm-1) was monitored for 30 min (reflecting the formation of thionitrobenzoate).

### Western blotting

The protein contents of COX17, COX11, SCO2, and COX-IV were determined by Western blot. Cells scraped in PBS were washed 3 times and the cell lysate was collected by using 1% SDS solution. Protein samples were mixed with 5× loading buffer, boiled for 10 min at 100°C and cooled. Equal amounts of protein (50–100 µg) from each sample were separated by 12% SDS-PAGE. Proteins were then electrophoretically transferred to a polyvinylidene fluoride membrane (Bio-Rad, USA). Transferred proteins were blocked with 5% non-fat dry milk in Tris-HCl buffer solution containing Tris-HCl (50 mM), NaCl (150 mM), and Tween-20 (0.1%) (TBS-T) for 1 h at room temperature. The blots were then incubated with respective primary antibodies (anti-COX17, anti-COX11, and anti-COX-IV, Santa Cruz, USA; anti-SCO2, Abcam, USA) in blocking solution according to the vender's recommendations. After incubation, the blots were washed with TBS-T six times for 5 min each. The blots were incubated for 2 h with appropriate secondary antibody. After washing six times (5 min each), target proteins were visualized using chemiluminescence (Bio-rad, USA) and analyzed by densitometry using a Quantity One Software.

### Statistical analysis

Data were obtained from three separate experiments and expressed as means ± S.E.M. A multiple factorial design was applied to this study. After a significant interaction was detected by the analysis of variance (ANOVA), the significance of the main effects was further determined by F-test. The level of significance was considered when *P*<0.05.

## Results

### PE-induced elevation of the intracellular and extracellular homocysteine concentrations

**Figure 1:**
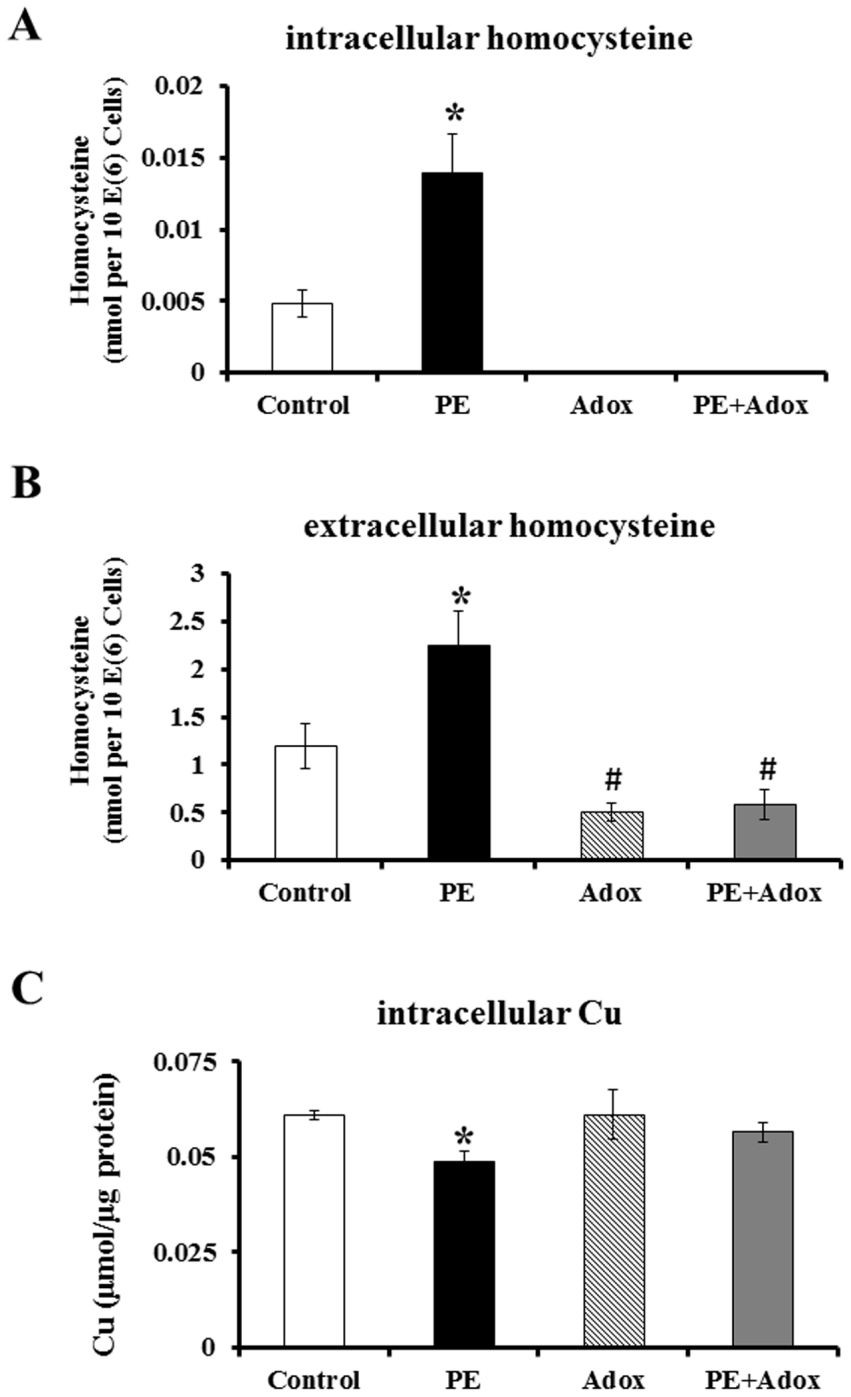
PE-induced changes in the intracellular (A) and extracellular homocysteine concentrations (B), and changes in the intracellular Cu concentrations (C) in primary cultures of neonatal rat cardiomyocytes. The cells were cultured in 10% FBS media for 24 h before medium change to serum-free media. The cells were then cultured for 72 h (Control), in the presence of 100 µM PE (PE), 1 µM Adox (Adox), or both PE and Adox (PE+Adox). The data were obtained from three independent experiments, and each experiment contains triplicate samples for each treatment. Values are means ± SEM. * or # significantly different from control group and # significantly different from PE-treated group (P<0.05).

Cardiomyocyte hypertrophy, measured by cell size, cellular protein contents, molecular markers of the hypertrophy, was observed in the primary cultures of neonatal rat cardiomyocytes treated with 100 µM PE for 48 hrs (Supplement 1). In the hypertrophic cardiomyocytes, homocysteine levels were significantly increased (Fig 1A). A parallel increase in the extracellular homocysteine concentrations was observed, as determined by the elevation of homocysteine concentrations in the culture media (Fig 1B). Homocysteine concentrations in the stock media were undetectable and in the media obtained from cultures without PE treatment were 1.2 µmol/L and increased to 2.2 umol/L after PE treatment. With the increase in both intracellular and extracellular homocysteine concentrations, the intracellular Cu concentrations were significantly decreased (Fig 1C). Addition of Adox, which inhibits S-adenosyl-L-homocysteine hydrolase (SAHH) in homocysteine synthesis, significantly reduced both intracellular (Fig 1A) and extracellular (Fig 1B) homocysteine concentrations, and blocked the PE-induced elevation. The inhibition of the intracellular homocysteine synthesis prevented PE-induced decrease in the intracellular Cu concentrations (Fig 1C).

### The effect of inhibition of homocysteine synthesis on PE-induced CCO depression

**Figure 2:**
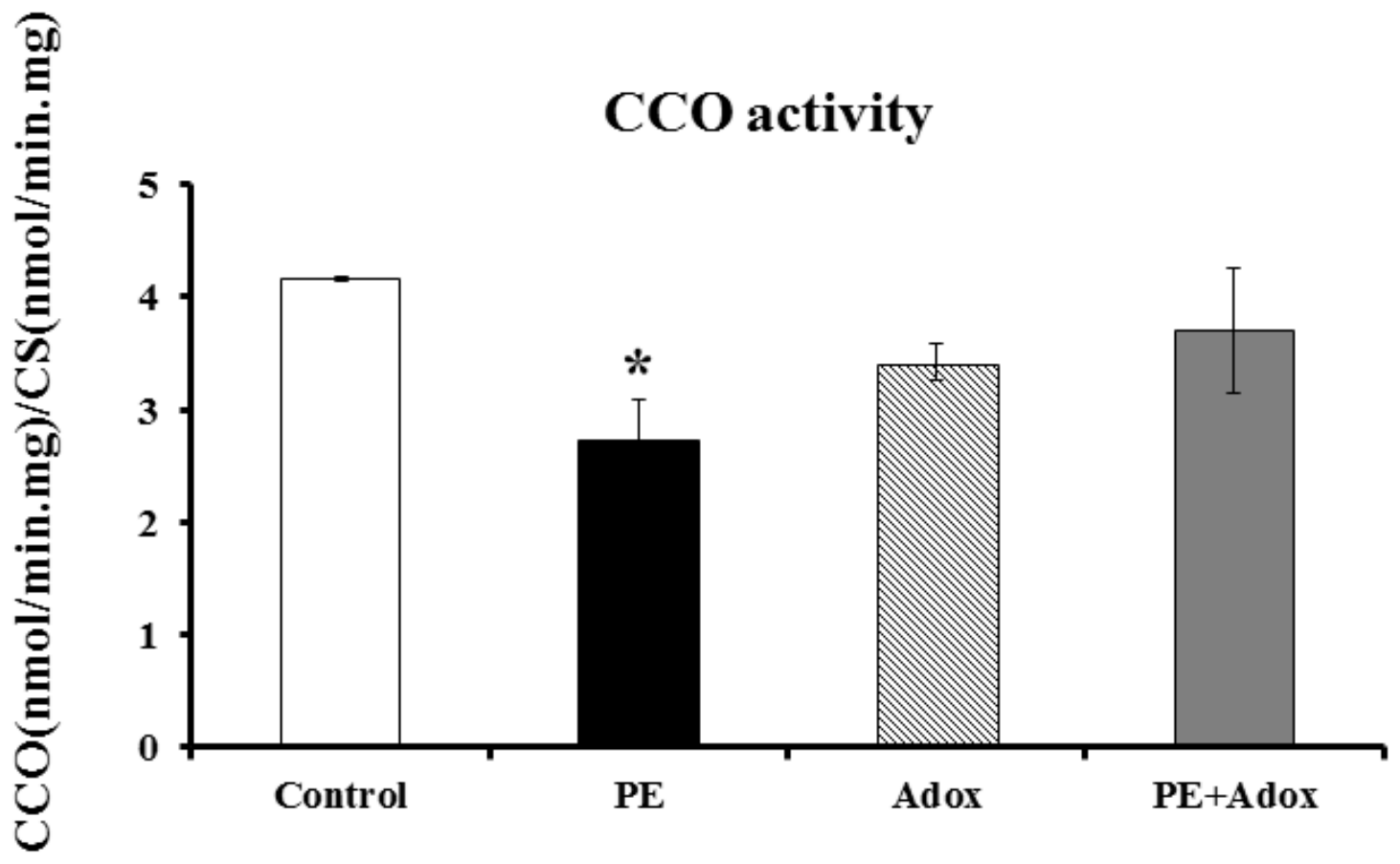
Changes in the CCO activity in the PE-treated primary cultures of neonatal rat cardiomyocytes. The cells were cultured and treated as the same as described for Fig 1. The data were obtained from three independent experiments, and each experiment contains triplicate samples for each treatment. Values are means ± SEM. * significantly different from control group (P<0.05).

In the PE-treated cardiomyocytes, the CCO activity was significantly depressed, as shown in Fig 2. Addition of SAHH inhibitor Adox alone did not significantly affect the CCO activity, but blocked PE-induced depression of the CCO activity in the primary cultures of neonatal rat cardiomyocytes.

### PE decreased protein levels of CCO subunit COX-IV as well as Cu chaperones COX17, COX11, and SCO2

**Figure 3:**
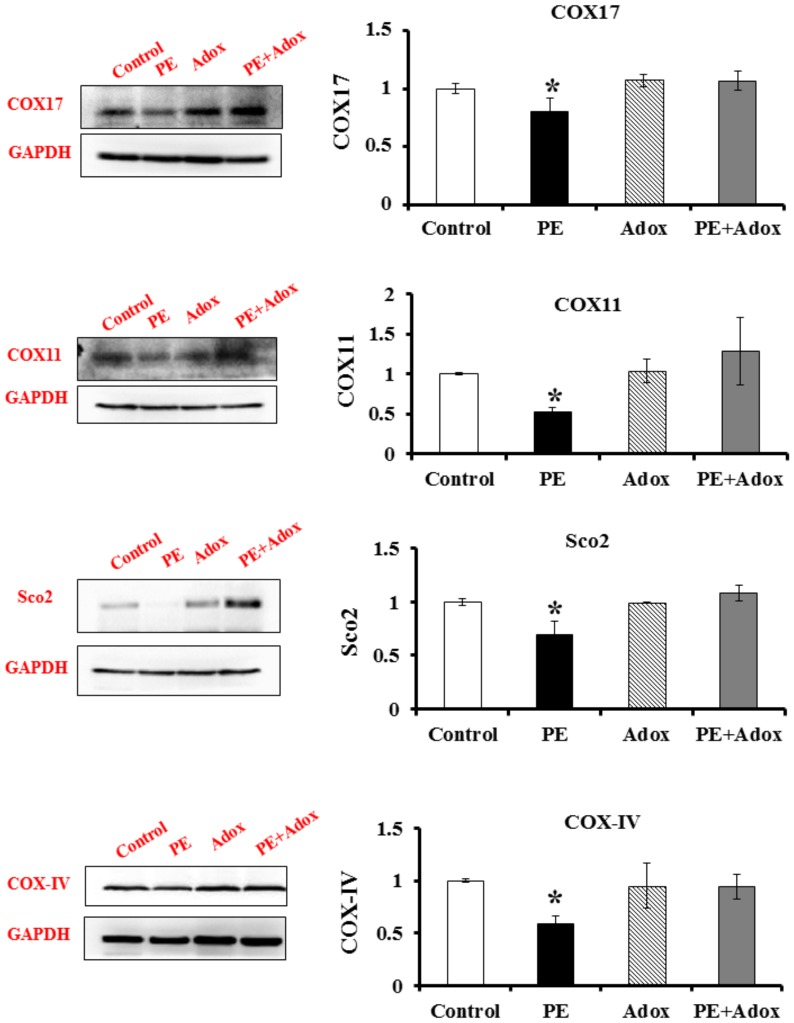
Immunoblots of COX17, COX11, SCO2 and COX-IV proteins from PE-treated primary cultures of neonatal rat cardiomyocytes and the effect of Adox. The cells were cultured and treated as the same as described for Fig 1. The bar graphs show the quantitative analysis of the intensity of immunoblots by densitometry. Values are means ± SEM. * significantly different from control group (P<0.05).

To understand the molecular mechanism by which homocysteine suppresses the CCO activity, the protein concentrations of COX17, COX11, SCO2, and COX-IV were measured by Western blot analysis. The data presented in Fig 3 showed that PE significantly reduced the concentrations of all the proteins measured. Although the SAHH inhibitor Adox did not affect the concentrations of these proteins, it prevented their reduction by PE treatment.

### Cu supplement prevented PE-induced depression of COX17, COX11, SCO2, and COX-IV

**Figure 4:**
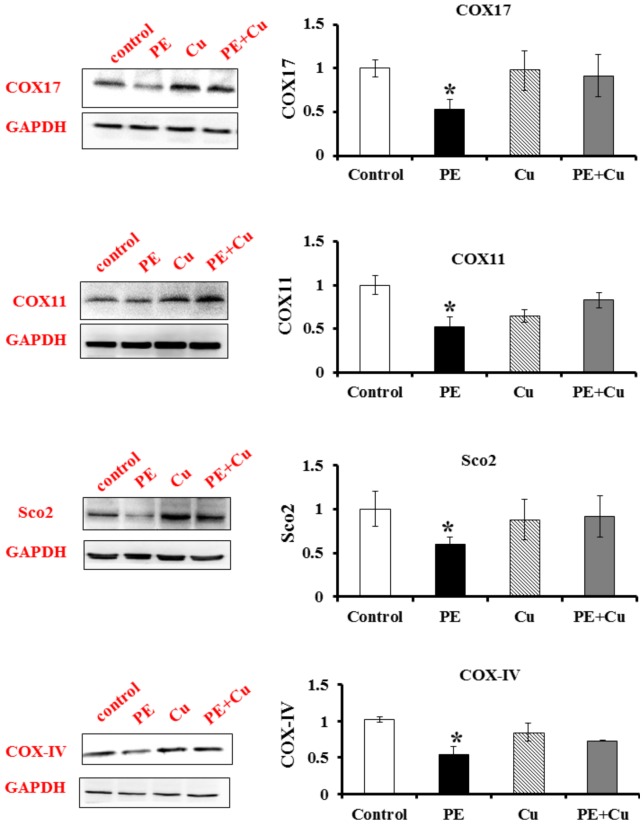
Immunoblots of COX17, COX11, SCO2 and COX-IV proteins from the PE treated primary cultures of neonatal rat cardiomyocytes. Control (cultured for 72 h with non-PE or other treatment), PE (PE treated for 72 h), Cu (cultured without treatment for 48 h then treated with Cu for 24 h), and PE+Cu (PE treated for 48 h followed by Cu treatment for 24 h). The bar graphs show the quantitative analysis of the intensity of immunoblots by densitometry. Values are means ± SEM. * significantly different from control group (P<0.05).

To determine whether the suppression by homocysteine of the CCO activity acts through the restriction of Cu availability, the cell cultures were supplemented with CuSO_4_. The addition of a final concentration of Cu element at 5 µM in the culture completely blocked the PE-induced reduction in the protein concentrations of CCO assembly subunit COX-IV and Cu chaperones COX17, COX11 and SCO2 (Fig 4).

## Discussion

The results presented here specifically addressed the mechanism by which PE treatment suppresses the CCO activity in cardiomyocytes. Cu is required for the CCO activity, and conversely, Cu deficiency leads to the suppression of the CCO activity [11]–[13]. In pressure overload-induced hypertrophic hearts and in PE-induced hypertrophic cardiomyocytes, Cu supplementation recovers the suppressed CCO activity and reverses the hypertrophy [23], [24]. Therefore, it is conclusive that Cu limitation results in suppression in the CCO activity in hypertrophic cardiomyocytes. However, there are two specific questions that have not been addressed: (1) how does Cu restriction take place in the hypertrophic cardiomyocytes, and (2) what is the molecular basis for the suppression of the CCO activity?

In the present study, we found that PE treatment resulted in an enhanced production of homocysteine in the cultured neonatal rat cardiomyocytes, leading to increases in both intracellular and extracellular concentrations of homocysteine. On equal cell number basis, the extracellular homocysteine concentrations were 100 fold higher than the intracellular concentrations. The level of homocysteine in the stock media was undetectable, thus, the extracellular concentrations were likely exported from the cells. This was strongly supported by the fact that inhibition of the intracellular homocysteine synthesis by using the SAHH inhibitor Adox reduced the extracellular concentrations of homocysteine to the level even below that found in the culture media without PE treatment.

The increase in the intracellular homocysteine was accompanied by a decrease in the intracellular Cu concentrations. The link between these two events was revealed by the prevention of the decrease in Cu concentrations by the inhibition of homocysteine synthesis in the PE-treated cardiomyocytes. This thus suggests that the reduction in Cu concentrations and its availability is related to the enhanced homocysteine production. How does homocysteine limit the availability of intracellular Cu? There are reports demonstrating the formation of Cu and homocysteine complexes in *in vitro* studies [30]-[32]. We also detected the Cu-homocysteine complexes when they were mixed *in vitro* in another study (unpublished). This, in combination with the result above, answers the first question, i.e., PE-induced elevation of intracellular homocysteine concentrations is responsible for Cu restriction in the hypertrophic cardiomyocytes.

The suppression of the CCO activity in the PE-induced hypertrophic cardiomyocytes resulted from the elevation of intracellular homocysteine. This was demonstrated by the fact that the inhibition of homocysteine synthesis blocked the suppression by PE treatment of the CCO activity. What is the molecular basis for the suppression of the CCO activity? The assembly of the 13 subunits of CCO is an important process for the activation of the enzyme. During this process, it is crucial for Cu transfer by Cu chaperones for CCO to the Cu active sites on the mitochondrial-encoded subunits COX-I and COX-II. It was expected that the reduction in Cu concentrations would restrict the availability of Cu to the Cu active sites. However, this restriction apparently is only one factor responsible for the suppressed CCO activity. The results presented in this study demonstrated that the impairment of the CCO assembly due to the reduction of COX-IV subunit would be another determinant factor. In addition, the disturbance of Cu transfer to Cu active sites in the CCO due to the reduction of the Cu chaperones COX17, COX11 and SCO2 would worsen the impairment of the CCO assembly. The contribution of the impairment of the CCO assembly to the suppressed CCO activity was further confirmed by the results that the inhibition of homocysteine synthesis prevented the reduction of COX-IV, COX17, COX11, and SCO2, and preserved the CCO activity. Therefore, the answer to the second question is that the suppressed CCO activity results at least in part from the impaired assembly of the holo-CCO.

How does the elevation of homocysteine affect the CCO assembly? We hypothesized that the restriction of Cu availability is responsible for the observed effect of homocysteine on the CCO assembly. To test this, we added physiologically relevant levels of Cu to the PE-treated cultures to examine the effect of Cu supplementation on PE-reduced concentrations of proteins involved in the CCO assembly. Under this condition, the elevation of homocysteine would not be affected, but the availability of Cu would be increased. The data obtained showed that Cu supplementation prevented the reduction of COX-IV, COX17, COX11, and SCO2 protein concentrations along with the preservation of the CCO activity. These results thus indicate that the reduced Cu availability or the Cu restriction is responsible for the suppressed CCO assembly induced by the elevation of homocysteine concentrations.

We speculate that the interaction between Cu and homocysteine, or the formation of Cu and homocysteine complexes is responsible for the restriction of Cu availability. This was also proposed in an early study examining homocysteine-induced CCO depression and apoptosis in neural cells [36], which stated that the binding of Cu and homocysteine would be responsible for the adverse effects of homocysteine. Although the formation of Cu and homocysteine complexes has been detected in *in vitro* studies, it is important to detect these complexes *in vivo* in future studies.

A limitation of the present study is that the mechanism by which PE treatment increases homocysteine production remains unknown. One of the pathways for the metabolism of homocysteine is its transsulfuration catalyzed by cystathionine β-synthase to become cystathionine, which in turn is converted to cysteine and α-ketobutyrate by cystathionine γ- lyase. It has been shown that oxidative inhibition of cystathionine β-synthase occurs, leading to accumulation of intracellular homocysteine [37]. Previous studies have demonstrated the production and accumulation of reactive oxygen species PE in cardiomyocytes [38]. Therefore, it is possible that PE-induced increase of homocysteine concentrations may result from the oxidative inhibition of the cystathionine β-synthase, which will be determined in our future studies.

In conclusion, the present study addressed the role of homocysteine in PE-suppressed CCO activity in hypertrophic cardiomyocyte. Homocysteine restricts the availability of intracellular Cu through its interaction with Cu and the suppression of Cu chaperones for CCO and CCO assembly subunits, leading to inhibition of the CCO activity. Conversely, inhibition of intracellular homocysteine synthesis results in preservation of the CCO activity and prevention of the subsequent adverse effects.

## Supporting Information

Figure S1 **PE-induced cell hypertrophy in primary cultures of neonatal rat cardiomyocytes.** The cells were cultured in 10% FBS media for 24 h before medium change to serum-free media, culturing for 72 h. (**A**) Analysis of cell size by flow cytometry with a representative histograph and quantitative measurement. Control (non-treated and incubated for 72 h), PE (PE treated for 72 h). (**B**) Changes in total protein concentrations, normalized by cell number. (**C**) Changes in the expression of β-MHC, α-SA, and ANP, measured by real-time RT-PCR. Each group of data was obtained from three independent experiments, and each experiment contains triplicate samples for each treatment. Values are means ± SEM. *significantly different from control group (P<0.05).
(TIF)
