# Supplementary figures and images for: Homocysteine Restricts Copper Availability Leading to Suppression of Cytochrome C Oxidase Activity in Phenylephrine-Treated Cardiomyocytes
Source: PLoS One. 2013 Jun 20;8(6):e67549. doi: 10.1371/journal.pone.0067549 (PMC3688604; doi:10.1371/journal.pone.0067549)

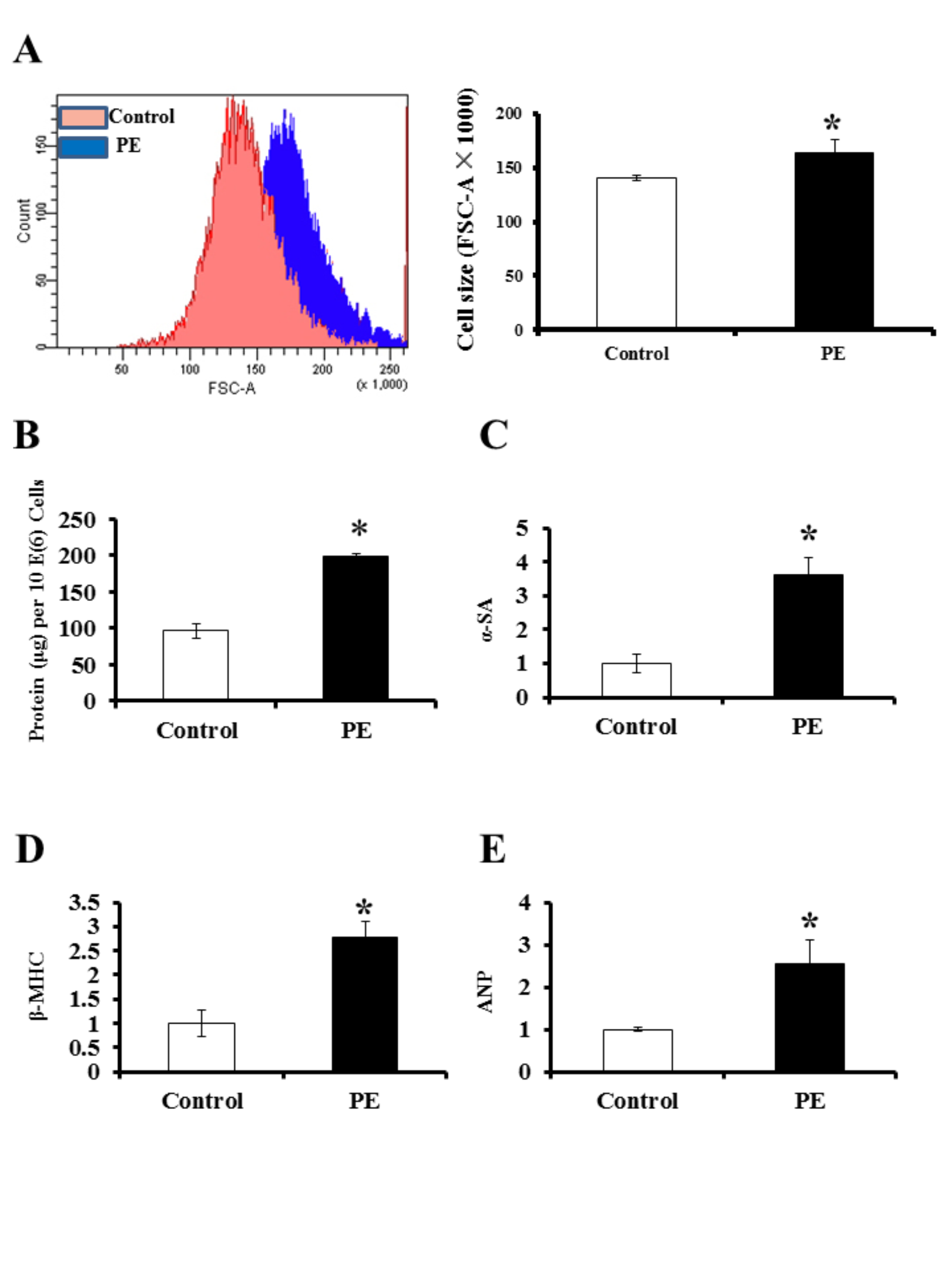

Supplement: Figure S1 — PE-induced cell hypertrophy in primary cultures of neonatal rat cardiomyocytes. The cells were cultured in 10% FBS media for 24 h before medium change to serum-free media, culturing for 72 h. (A) Analysis of cell size by flow cytometry with a representative histograph and quantitative measurement. Control (non-treated and incubated for 72 h), PE (PE treated for 72 h). (B) Changes in total protein concentrations, normalized by cell number. (C) Changes in the expression of β-MHC, α-SA, and ANP, measured by real-time RT-PCR. Each group of data was obtained from three independent experiments, and each experiment contains triplicate samples for each treatment. Values are means ± SEM. *significantly different from control group (P<0.05). (TIF) [file pone.0067549.s001.tif]
